# Prolonged elevation of serum neurofilament light after concussion in male Australian football players

**DOI:** 10.1186/s40364-020-00256-7

**Published:** 2021-01-10

**Authors:** Stuart J. McDonald, William T. O’Brien, Georgia F. Symons, Zhibin Chen, Jesse Bain, Brendan P. Major, Daniel Costello, Glenn Yamakawa, Mujun Sun, Rhys D. Brady, Biswadev Mitra, Richelle Mychasiuk, Terence J. O’Brien, Sandy R. Shultz

**Affiliations:** 1 Department of Neuroscience, Central Clinical School, Monash University, Melbourne, VIC Australia; 2 Department of Physiology, Anatomy, and Microbiology, La Trobe University, Melbourne, VIC Australia; 3 Clinical Epidemiology, Monash University, Melbourne, VIC Australia; 4 Department of Medicine, The University of Melbourne, Parkville, VIC Australia; 5 National Trauma Research Institute, Melbourne, VIC Australia; 6 Emergency and Trauma Centre, The Alfred Hospital, Melbourne, VIC Australia; 7 Department of Epidemiology and Preventive Medicine, Monash University, Melbourne, VIC Australia

**Keywords:** Mild traumatic brain injury, Sports-related concussion, Blood biomarker, Diagnosis, Recovery, Return to play, GFAP, Tau, UCHL1, NfL

## Abstract

**Background:**

Biomarkers that can objectively guide the diagnosis of sports-related concussion, and consequent return-to-play decisions, are urgently needed. In this study, we aimed to determine the temporal profile and diagnostic ability of serum levels of neurofilament light (NfL), ubiquitin carboxy-terminal hydrolase L1 (UCHL1), glial fibrillary acidic protein (GFAP), and tau in concussed male and female Australian footballers.

**Methods:**

Blood was collected from 28 Australian rules footballers (20 males, 8 females) at 2-, 6-, and 13-days after a diagnosed concussion for comparison to their levels at baseline (i.e. pre-season), and with 27 control players (19 males, 8 females) without a diagnosis of concussion. Serum concentrations of protein markers associated with damage to neurons (UCHL1), axons (NfL, tau), and astrocytes (GFAP) were quantified using a Simoa HD-X Analyzer. Biomarker levels for concussed players were compared over time and between sex using generalised linear mixed effect models, and diagnostic performance was assessed using area under the receiver operating characteristic curve (AUROC) analysis.

**Results:**

Serum NfL was increased from baseline in male footballers at 6- and 13-days post-concussion. GFAP and tau were increased in male footballers with concussion at 2- and 13-days respectively. NfL concentrations discriminated between concussed and non-concussed male footballers at all time-points (AUROC: 2d = 0.73, 6d = 0.85, 13d = 0.79), with tau also demonstrating utility at 13d (AUROC = 0.72). No biomarker differences were observed in female footballers after concussion.

**Conclusions:**

Serum NfL may be a useful biomarker for the acute and sub-acute diagnosis of concussion in males, and could inform neurobiological recovery and return-to-play decisions. Future adequately powered studies are still needed to investigate biomarker changes in concussed females.

**Supplementary Information:**

The online version contains supplementary material available at 10.1186/s40364-020-00256-7.

## Background

Despite increasing awareness of the risks associated with sports-related concussion (SRC) [1], the identification and management of this condition remains notoriously difficult [2–4]. Blood biomarkers have potential to provide reliable and sensitive tests to aid in SRC diagnosis and determination of brain recovery (e.g. return to play, RTP) [5]. Although some recent studies have indicated potential of biomarker candidates in SRC, particularly when measured in the hours immediately following injury, overall findings to date have been mixed [6–12]. Several factors have likely contributed to the variable results, including single or disparate collection time-points, differences in analytical methods, and a lack of baseline (e.g. pre-season) or non-concussed controls. Two recent studies that featured serial sampling with appropriate control groups have indicated some diagnostic utility of neurofilament light (NfL), tau, and glial fibrillary acid protein (GFAP), however findings differed between the studies [6, 7]. Additionally, markers were primarily assessed in the acute stages and at non-fixed time-points in the weeks following injury. As such, further studies are required to understand the temporal profile and utility of these biomarker candidates beyond the acute stages of SRC. This is an important knowledge gap, as many athletes with suspected concussion do not immediately seek medical attention [13, 14]. Moreover, as studies to date have primarily focused on male athletes, little is known about how biological sex may affect biomarker profiles. Accordingly, this prospective current study of male and female Australian footballers aimed to compare serum concentrations of NfL, tau, ubiquitin carboxy-terminal hydrolase L1 (UCHL1), and GFAP at 2-, 6-, and 13-days after SRC with levels at baseline and with control footballers without SRC.

## Methods

### Study population and study design

A total of 127 (82 males, 45 females) amateur Australian rules football players were recruited from the University of Melbourne Football Club (male collection period 2016–2019, female 2018–2019). The Melbourne Health Human Ethics committee approved study procedures, and all participants provided written informed consent. Australian football is the most participated collision sport in Australia, and both the men’s and women’s leagues follow similar full collision rules, which provides the opportunity to investigate sex differences within the same collision sport [15, 16].

**Fig. 1:**
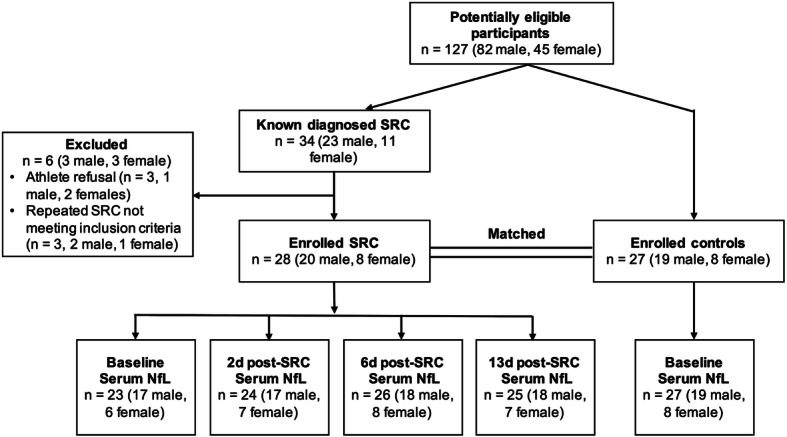
Flow chart for player recruitment. Of the 127 male and female Australian footballers enrolled in the study, 34 cases of diagnosed SRC were made known to the research team. After exclusion of 6 players, 28 cases of SRC were assessed for SCAT and serum biomarker profiles across time (i.e. baseline, 2-, 6- and 13-days post-SRC), and diagnostic accuracy (i.e. AUROC analysis) at each post-SRC timepoint was assessed against matched control footballers (see Table 1) without SRC

A matched prospective cohort study was conducted. Baseline samples were collected from footballers during each pre-season. Players who self-reported a diagnosis of concussion in the preceding 6 months, had a history of neurosurgery or severe psychiatric disturbances were excluded. Twenty-eight players (20 males, 8 females) were diagnosed by a team physician with an SRC during match play, and subsequently underwent blood sampling at 2-, 6- and 13-days. All within-subject comparisons were made with baseline samples from the corresponding pre-season. Five players (3 males, 2 females) who sustained an SRC during this period did not have this baseline sample and were included in the dataset, and a small number of post-SRC collections were missed due to logistical reasons (e.g. participant unavailability), resulting in a dataset of 23–26 samples per time-point (Fig. 1). An additional 27 baseline samples (19 males, 8 females) from closely matched players that did not sustain a concussion were selected by researchers blinded to biomarker data for use as controls for comparison with SRC samples (Table 1).

**Table 1:** Baseline demographic results for male and female Australian rules footballers. Players who sustained a concussion during the collection period were allocated into the SRC group, with non-concussed footballers serving as controls for each sex. Results are presented as Mean ± SD

	Male footballers			Female footballers		
	SRC	Control	***P***-value	SRC	Control	P-value
**N**	20	19	–	8	8	–
**Age**	23.8 ± 3.5	23.9 ± 3.1	0.85	24.6 ± 4.1	23.5 ± 3.4	0.56
**Years of education**	16.6 ± 2.2	16.9 ± 1.4	0.54	15.9 ± 2.2	15.5 ± 2.1	0.73
**Years of sport**	16.1 ± 5.0	17.5 ± 3.5	0.33	18.4 ± 5.4	16.1 ± 5.9	0.44
**Years of collision sport**	13.9 ± 4.6	14.4 ± 4.6	0.78	8.4 ± 5.7	7.4 ± 5.2	0.72
**No. of previous concussions**	2.1 ± 1.8	2.3 ± 1.7	0.78	0.1 ± 0.4	1.3 ± 1.8	0.13

### Clinical interview and symptom evaluation

A questionnaire was administered pertaining to factors including demographics, sporting history, education history, and history of concussion. The ‘sport concussion assessment tool’ (SCAT3 for 2017/18 players, SCAT5 for 2019 players) assessed symptom number (maximum 22) and severity (maximum 132) at baseline, and at 2-, 6-, and 13-days after SRC. Analysis of the cognitive component of the SCAT3 (‘standardized concussion assessment’, SAC; maximum 30) was performed for 2017/18 SRC participants.

### Blood collection

Using standard phlebotomy procedures, 20 mL of blood was collected into BD Vacutainer® SST™ II Advance tubes for serum preparation. The tube was inverted several times and allowed to clot at room temperature for 30 min prior to centrifugation at 1500 g for 10 min to separate serum. Serum was then transferred into aliquots, flash-frozen, and stored at − 80 °C.

### Serum analysis

Quantification of NfL, tau, UCHL1, and GFAP was performed using ‘Neurology 4-plex B’ kits run on the Simoa HD-X Analyzer (Quanterix, Billerica, MA, USA). All assays were performed within a one-week period using kits with the same lot number, in a temperature-controlled laboratory by an experimenter blinded to the clinical information. All samples were tested in duplicate, with a total volume for each sample of 80 μL. To minimise any potential batch effects, longitudinal samples from the same individual were run on the same plate, and groups were equally distributed between plates. All samples measured above the lower limit of quantification for NfL (0.500 pg/mL), GFAP (9.38 pg/mL) and tau (0.125 pg/mL). For UCHL1, 46/125 samples were found to be below the lower limit of detection and were therefore assigned this value (1.90 pg/mL). This was expected, as the UCHL1 portion of the Neurology 4-Plex B assay was optimized for specificity (rather than sensitivity) to best discriminate between healthy patients and those with traumatic brain injury. The average inter-plate coefficient of variation (CV) of controls samples for NfL, GFAP, tau, UCHL1 was 6, 4, 17, and 8%, respectively. The average intra-plate CV of samples for NfL, GFAP, tau, UCHL1 was 7, 7, 16, and 31%, respectively. These CVs are consistent with other studies utilizing this assay [17, 18].

### Statistics

Demographic variables were compared between SRC and non-SRC footballers of each sex with an unpaired t-test. Baseline measures of serum biomarker levels between SRC and non-SRC footballers for each sex were compared using unpaired t-tests or Mann-Whitney tests. Generalised linear mixed effect models with unstructured covariance structure were used to compare the change in mean values of the outcome variables (i.e. NfL, tau, UCHL1, GFAP, symptom number and severity, and SAC score) over time and between sex. Given the nature of the data, log-normal distribution was applied for NfL and GFAP, gamma distribution for tau and UCHL1, negative binomial distribution for symptom number and severity, and normal distribution for SAC score. The relationships between symptom measures (i.e., number and severity) and biomarker levels were assessed using Spearman’s correlation analysis. Area under the receiver operating characteristics (AUROCs) were estimated for each protein outcome (i.e. NfL, tau, UCHL1, and GFAP) at each time-point and sex in classifying participants with concussion or controls without concussion. Holm-Bonferroni method was applied to correct for multiple comparisons. Statistical significance was set at *p*< 0.05. All statistical tests were performed using GraphPad Prism version 8.10 (GraphPad Software Inc., San Diego, CA, USA) and Stata version 16 (StataCorp, College Station, TX, USA).

### Data availability

Data sharing requests will be considered by the corresponding author.

## Results

### Demographics

When comparing footballers in the SRC and non-SRC groups, there were no significant within-sex differences for self-reported age, total years of education, total sport and collision sport participation, nor history of concussion (Table 1).

### Clinical measures

**Fig. 2:**
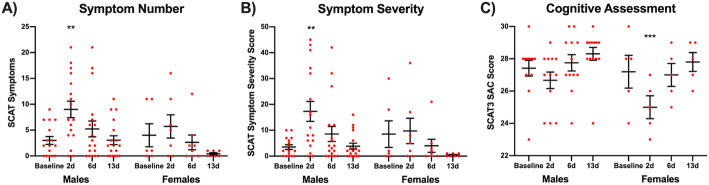
Concussion-related symptoms in male and female footballers. SCAT measures of symptom number **(a)** and symptom severity **(b)** were both increased in male footballers at 2-days after SRC when compared to pre-season baseline (**p=0.006). No differences in symptom number and severity compared to baseline were found at 6- and 13-days in males, and female footballers diagnosed with SRC, between baseline and 2-, 6- and 13-days after SRC. **c** Female footballers overall scored less on the SAC component of the SCAT3 at 2-days when compared to baseline (***p< 0.001). Individual participant results are plotted (red), along the group mean ± SEM. Note that symptom data was transformed for statistical analysis as described in the methods section

There were significant sex-time interaction effects for both symptom number (χ^2^=8.15, *p*=0.043) and symptom severity (χ^2^=10.6, *p*=0.014). The main effects of time were significant for both symptom number (χ^2^=49.3, *p*< 0.001) and symptom severity (χ^2^=66.7, *p*< 0.001), but the main effects of sex were not significant for both symptom number (χ^2^=2.64, *p*=0.104) and severity (χ^2^=1.98, *p*=0.160). Post-hoc analyses of results among male players revealed an increase in symptom number (contrast=6.33, 95% confidence interval [CI]: 2.35–10.3, *p*=0.006; Fig. 2a) and severity (contrast=13.4, 95% CI: 4.87–22.0, *p*=0.006; Fig. 2b) at 2-days post-SRC when compared to baseline (i.e. pre-season), with no differences from baseline found at 6- and 13-days. Post-hoc analysis within female SRC cases revealed no differences for symptom number and severity at all time-points.

For athletes that completed the SAC cognitive assessment (i.e. 2017/18 footballers), the main effect of sex and sex-time interaction were not significant, however there was a significant main effect of time (χ^2^=43.9, *p*< 0.001). Post-hoc analyses of results among female players revealed a significant decrease in SAC score at 2-days post-SRC when compared to baseline (contrast=− 2.20, 95% CI: − 3.25–-1.15, *p*< 0.001; Fig. 2c), but no differences from baseline found at 6- and 13-days. On the other hand, no significant differences in SAC scores were found among male players at any time points. All comparisons available in Supplementary Table 1.

### Temporal profile of serum biomarkers

**Fig. 3:**
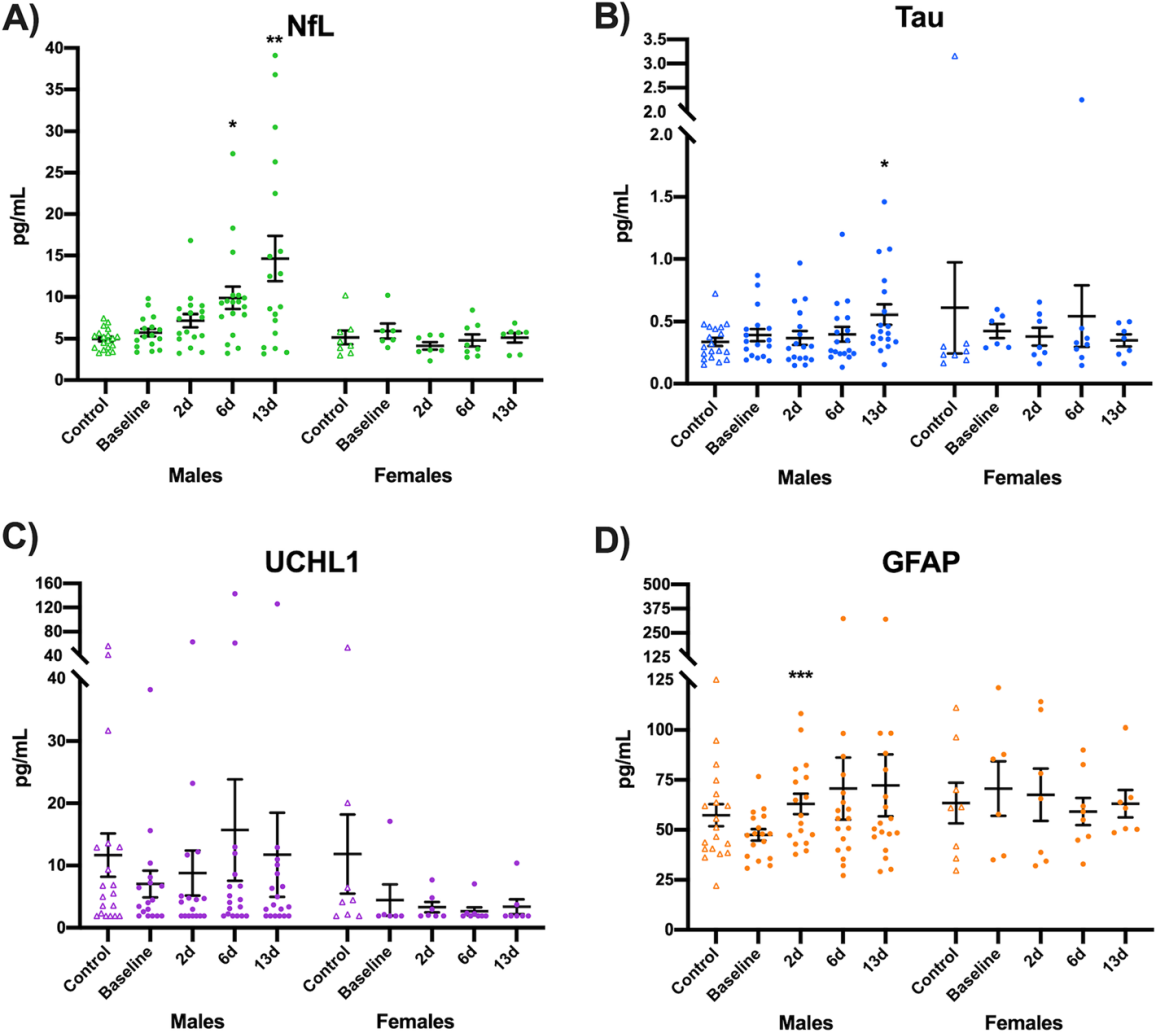
Temporal profiles of serum biomarkers in footballers with SRC. **a** Serum concentrations of NfL were increased from baseline (i.e. pre-season) at 6-days (*p=0.012) and 13-days (**p=0.003) after SRC in males. **b** Tau concentrations in serum were elevated at 13-days compared to baseline in male footballers (*p=0.039). **c** No changes in UCHL1 levels where found after SRC. **d** Serum GFAP was elevated at 2-days after SRC in males (***p< 0.001). No differences in serum NfL, tau, UCHL1 and tau were found in females diagnosed with SRC. Individual participant results are plotted, along the group mean ± SEM. Non-concussed controls are included as point of reference, and were not included in this statistical analysis. Note that serum biomarker data was transformed for statistical analysis as described in the methods

For NfL, a significant main effect of sex (χ^2^=20.5, *p*< 0.001) and sex-time interaction (χ^2^=17.9, *p*< 0.001) were found, while the main effect of time was not significant (χ^2^=4.49, *p*=0.21). Multiple comparisons of serum NfL in male footballers with SRC found an increase from baseline (i.e. pre-season) at 6-days (contrast=4.30, 95% CI: 1.21–7.38, *p*=0.012; Fig. 3a) and 13-days (contrast=8.64, 95% CI: 3.42–13.9, *p*=0.003), but not significantly increased at 2-days (contrast=1.52, 95% CI: − 0.17–3.20, *p*=0.078). No significant differences in serum NfL were found within females with SRC.

For tau, no significant main effect or interaction was found*.* However, serum tau was significantly increased in concussed male footballers at 13-days when compared to baseline (contrast=0.23, 95% CI: 0.05–0.42, *p*=0.039; Fig. 3b). No differences in tau were found at 2- and 6-days post-SRC in males, nor at any time-point within females.

For UCHL1, only main effect of sex was found to be significant (χ^2^=4.23, *p*=0.040), while main effect of time (χ^2^=3.03, *p*=0.39) and sex-time interaction (χ^2^=1.89, *p*=0.60) were both non-significant. Compared to the baseline, the UCHL1 was not changed at subsequent time-points in both males and females (Fig. 3c)*.*

For GFAP, the main effect of time (χ^2^=11.2, *p*=0.011) and sex-time interaction (χ^2^=8.13, *p*=0.043) were found to be significant, while the main effect of sex was not (χ^2^=0.430, *p*=0.51). Male footballers were found to have increased serum GFAP at 2-days compared to baseline (contrast=20.3, 95% CI: 9.66–30.9, *p*< 0.001; Fig. 3d), but not at 6-days after correction for multiple comparison (contrast=26.4, 95% CI: 1.10–51.7, *p*=0.082). Serum GFAP levels were not altered in females after SRC.

All aforementioned comparisons are available in Supplementary Table 1. No baseline differences were found between non-concussed control footballers and SRC footballers for all markers in both sexes (*p*> 0.05; Supplementary Table 2). Spaghetti plots of biomarker profiles are shown in Supplementary Fig. 1.

No correlations were found between serum biomarker levels and symptom measures at corresponding time-points post-SRC, with the exception of tau at 6-days in males (Symptom number, *r*=0.51, *p*=0.030; Symptom severity, *r*=0.51, *p*=0.033).

### Sensitivity and specificity of serum biomarkers

**Fig. 4:**
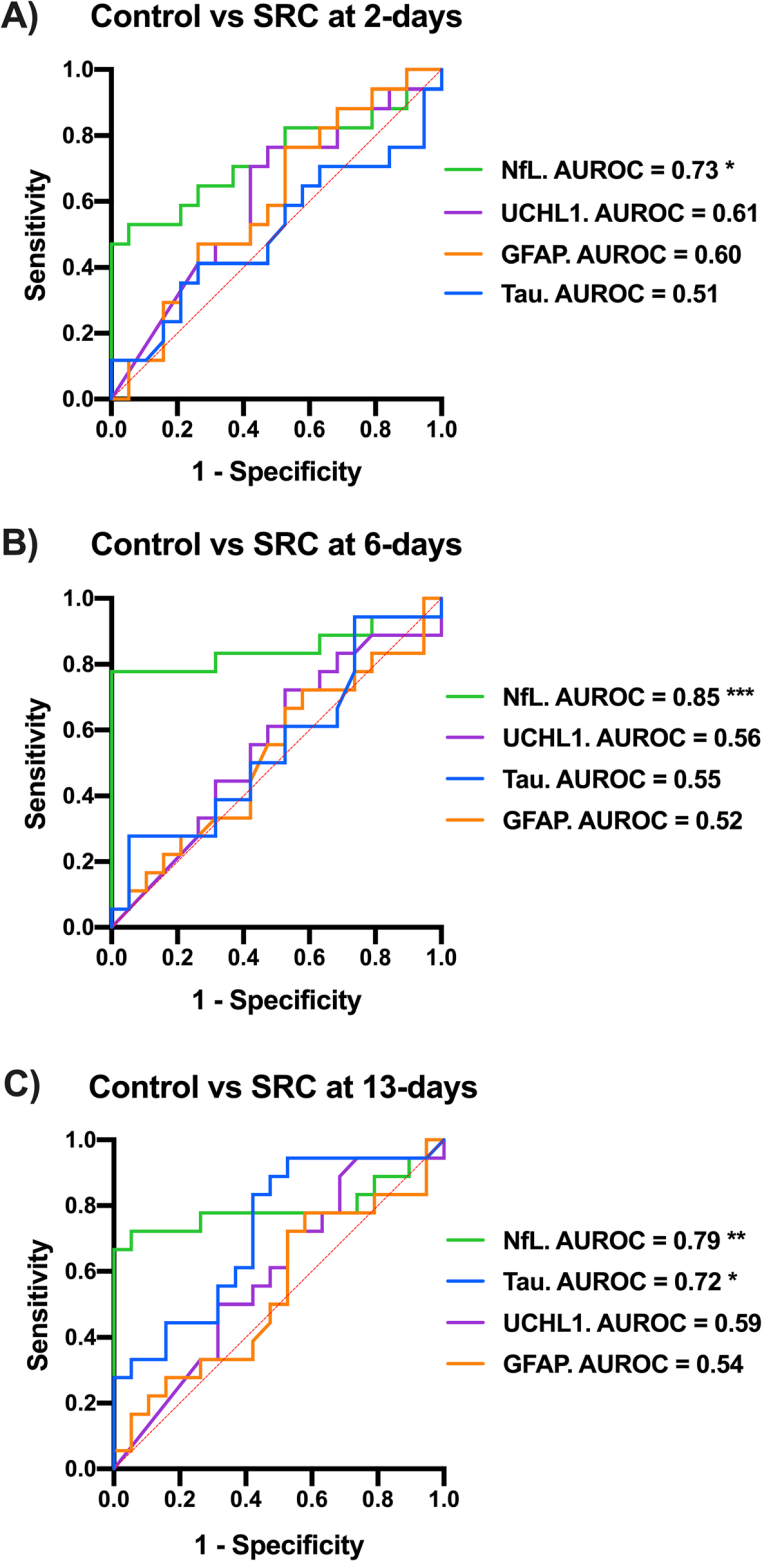
Sensitivity and specificity of serum biomarkers for SRC in males. Comparison of non-concussed (controls) with concussed male footballers with AUROC analysis revealed that: Only serum NfL could identify male players with SRC at 2-days (**a**; AUROC=0.73, *p=0.018) and 6-days (**b**; AUROC=0.85, ***p< 0.001). **c** At 13-days after SRC, serum NfL (AUROC=0.79; **p=0.002) and to a lesser extent serum tau (AUROC=0.72; *p=0.022) could distinguish male footballers with SRC

For each sex, AUROC analysis was used to compare the performance of serum biomarkers for distinguishing between non-concussed (control) footballers and footballers with SRC at 2-, 6- and 13-days post-SRC. In males, at 2-days serum NfL was able to distinguish SRC from control footballers with AUROC of 0.73 (*p*=0.018; Fig. 4a), whereas serum tau, UCHL1 and GFAP displayed no such diagnostic utility at this time-point. Serum NfL was able to distinguish male control footballers from male footballers with SRC at 6-days with an AUROC of 0.85 (*p*< 0.001; Fig. 4b), whereas serum levels tau, UCHL1 and GFAP could not. At 13-days post-SRC (Fig. 4c), both NfL (AUROC=0.79, *p*=0.002) and tau (AUROC=0.72, *p*=0.022) were able to distinguish male SRC from male control footballers, whereas no such utility was found for UCHL1 and GFAP. No differences were found between female control and SRC footballers at all time-points (*p*> 0.05; Supplementary Table 3).

## Discussion

The primary finding of this study was that SRC in male Australian footballers resulted in an elevation in serum NfL at 6- and 13-days when compared to pre-season baseline, with NfL levels at 2-, 6- and 13-days able to differentiate concussed and non-concussed male players. We also found elevations in GFAP and tau within males at 2- and 13-days after SRC respectively; however, only tau at 13d was able to differentiate concussed and non-concussed footballers. Biomarker differences were not observed among female footballers with SRC. Overall, these findings indicate that NfL may be useful as a blood biomarker to screen for concussion in male athletes, with the prolonged elevations indicating potential utility for assisting in concussion diagnosis at several time-points after injury, and possibly, for aiding in determination of recovery and RTP decisions.

### Blood changes in GFAP, NfL and tau after SRC

A strength of our study was the inclusion of pre-season baseline measures that allowed for comparisons of pre- and post-concussion serum protein levels, in addition to the cross-sectional comparisons with non-concussed control subjects that has been done in most previous studies. These within-subject comparisons of male footballers revealed no change in UCHL1, an acute increase in serum GFAP, a delayed increase in tau levels, and a sustained increase in NfL.

The temporal profiles for serum UCHL1 and GFAP are mostly in line with the limited literature on SRC and mild traumatic brain injury, with studies to date indicating UCHL1 increases are only seen acutely (i.e. within hours) and GFAP primarily within the first 24 h of injury [7, 19–21]. As such, it is possible that the peak expression of both UCHL1 and GFAP were missed in the current study. Although within-subject comparisons of males found that GFAP was increased at 2-days when compared to baseline, the inability to distinguish from non-concussed footballers limits the broader diagnostic utility of this biomarker at this time-point.

There is growing evidence that circulating increases of tau and NfL may occur in a biphasic manner after SRC [6, 7], potentially reflective of axonal damage due to primary and secondary injury processes [22, 23]. For example, a recent study found that when compared to unmatched pre-season samples and healthy controls, male hockey players had increases in both tau and NfL that appeared to peak at 1 h post-SRC, drop at 12 h, and rise again at 6-days [6]. Our findings of elevated tau (13-days) and NfL (6- and 13-days) provide evidence that assessment of axonal injury markers may have utility well into the subacute stages of SRC. Moreover, that serum NfL (all time-points) and tau (13-days) were able to distinguish between footballers with and without SRC indicates a potential utility of these markers even in the absence of baseline control samples. This is an important consideration in the ultimate translation of SRC biomarkers to the broader sporting community, where the feasibility of widespread baseline sampling is low.

### Blood biomarker changes were apparent beyond the resolution of SRC symptoms

SCAT assessments indicated that symptoms were apparent at 2-days, but not at 6- and 13-days post-SRC. As such, our findings of blood biomarker changes in males beyond 2-days provides further evidence that neurobiological recovery may take longer than clinical recovery [24–27]. Although it is possible that more sophisticated neuropsychological testing may have revealed symptoms or deficits that persisted beyond that measured in our study [28–30], the use of self-reported symptom reporting is often used as the primary outcome for determination of SRC recovery and RTP advice [27, 31]. Notably, all male players included in this study were cleared for RTP by their respective team clinician 14-days after SRC (i.e. the day following last blood sampling). The clinical significance of persistently elevated biomarkers beyond the period of symptoms remains unknown and a potential focus for follow-up. It is also important to note that, with the exception of tau in males at 6-days after SRC, no correlations were found between biomarker and symptom profiles. Such disparity provides further evidence that reliance on subjective self-reported symptoms alone may not provide an accurate reflection on the degree of injury and recovery.

Although it is important to consider factors such as the circulating half-life of tau and NfL, which are unknown but speculated to be relatively long for NfL [32], our data indicate ongoing release of these axonal injury markers into circulation well into the sub-acute stages of SRC. As such, although the mechanisms of the widely hypothesised ‘window of cerebral vulnerability’ following concussion are poorly understood [27, 33–36], it is possible that ongoing axonal injury or repair may represent a neurobiological factor that increases susceptibility to effects of repeated concussions. If proven to be the case, an additional utility of associated biomarkers such as tau and NfL may be in aiding determination of recovery and RTP advice. However, further research is still required to understand how these biomarkers reflect changes in the concussed brain, if they can indicate neurobiological recovery, and if their implementation in RTP decisions can mitigate the cumulative burden of repeated brain injuries. Animal models of concussion may be particularly useful in this context [37–39].

### Limitations and future directions

A limitation of the current study is the lack of objective measures to complement blood biomarker quantification. For example, high resolution diffusion MRI would have allowed white matter changes due to SRC to be localized and quantified, and provided supportive evidence that increases in serum NfL and tau were indeed reflective of axonal injury. As such, future multi-modal studies are required to determine if and how serum biomarker changes correlate with advanced neuroimaging metrics in the acute and sub-acute stages of SRC.

Despite increasing awareness that females may be more vulnerable to concussion than males [26, 40], to date only a small selection of SRC blood biomarker studies have incorporated female athletes [7, 8, 20]. Unexpectedly, we found no utility of serum NfL, tau, UCHL1, and GFAP following SRC in females. This lack of efficacy may have resulted from genuine biological sex differences in SRC biomarker profiles, sex differences in injury severity, or our limitation of a low sample size of females. Although not without limitation, SCAT symptom results may indeed indicate differences in injury severity between sexes, as only males had differences based on self-reported symptoms at 2-days after SRC. Nonetheless, it is important to note that both male and female footballers were diagnosed with SRC by a team clinician on match day. Furthermore, it is likely that self-reported symptoms would have been more pronounced had SCAT scores been analysed more acutely post-SRC rather than 2-days. Although SCAT tests have well-established utility for assisting SRC diagnosis in both sexes, it should be acknowledged that this assessment is not specifically designed for quantification of post-SRC symptoms. As such, the absence of increased self-reported symptoms at 2-days in females should not be overinterpreted, particularly considering that self-reported symptoms are widely acknowledged to have bias susceptibility [41–43], and some studies have found that females may report more concussion-related symptoms at baseline [44]. In addition, our findings indicated that only females had a reduction in SAC scores at 2-days when compared to baseline. As such, although reduced severity of injury in females is a biologically plausible explanation for the lack of changes in serum levels of biomarkers associated with axonal and glial damage, further studies incorporating additional indicators of injury (e.g. neuropsychological testing, neuroimaging) are required to provide additional insights. Taken together, future studies specifically designed and adequately powered to examine how these biomarkers, and others, are affected by sex are required.

## Conclusions

Serum NfL may aid in SRC diagnosis throughout the acute and sub-acute stages of injury in males, but further work is required for biomarkers of SRC in females. As our findings indicate that axonal injury may persist beyond symptom resolution and into the sub-acute stages of SRC, serum biomarkers such as NfL may prove to have utility for informing neurobiological recovery and RTP decisions.

## Supplementary Information

**Additional file 1: Supplementary Table 1.** SCAT and serum biomarker analyses of concussed male and female Australian football players. **Supplementary Table 2.** Baseline comparisons between non-SRC control footballers and SRC footballers. **Supplementary Table 3.** Serum biomarker comparisons of non-concussed and concussed Australian footballers. **Supplementary Figure 1.** Temporal profile of serum biomarkers pre- and post-concussion.

## Data Availability

The datasets used and/or analysed during the current study are available from the corresponding author on reasonable request.

## Abbreviations

AUROC: Area under the receiver operating characteristic curve
CI: Confidence Interval
CV: Coefficient of variation
GFAP: Glial fibrillary acidic protein
NfL: Neurofilament light
SAC: Standardized concussion assessment
SCAT: Sport concussion assessment tool
SRC: Sports-related concussion
RTP: Return to play
UCHL1: Ubiquitin carboxy-terminal hydrolase L1
