# Supplementary material for: Prolonged elevation of serum neurofilament light after concussion in male Australian football players
Source: Biomark Res. 2021 Jan 10;9:4. doi: 10.1186/s40364-020-00256-7 (PMC7797141; doi:10.1186/s40364-020-00256-7)
Supplement: Supplementary file 1 — Additional file 1: Supplementary Table 1. SCAT and serum biomarker analyses of concussed male and female Australian football players. Supplementary Table 2. Baseline comparisons between non-SRC control footballers and SRC footballers. Supplementary Table 3. Serum biomarker comparisons of non-concussed and concussed Australian footballers. Supplementary Figure 1. Temporal profile of serum biomarkers pre- and post-concussion. [file 40364_2020_256_MOESM1_ESM.docx]

**Supplementary Table 1.** SCAT and serum biomarker analyses of concussed male and female Australian football players

|  | **Main Effect and Interaction**  **(χ^2^, *p*-value)** | | | **Multiple Comparisons**  **(Contrast, 95% CI, Holm-Bonferroni Corrected *p*-value)** | | | |
| --- | --- | --- | --- | --- | --- | --- | --- |
|  | **Sex** | **Time** | **Interaction** |  | **Baseline vs 2-days** | **Baseline vs 6-days** | **Baseline vs 13-days** |
| **SCAT Symptom Number** | χ^2^=2.64, *p*=0.104 | **χ^2^=49.3, *p*<0.001** | **χ^2^=8.15, *p*=0.043** | Males | **Contrast=6.33,**  **95% CI: 2.35 – 10.3, *p*=0.006** | Contrast=2.89,  95% CI: -0.86 – 6.63, *p*=0.262 | Contrast=2.05,  95% CI: -2.90 – 7.00, *p*=0.417 |
|  |  |  |  | Females | Contrast=1.19,  95% CI: -3.41 – 5.80, *p*=0.612 | Contrast=-1.57,  95% CI: -5.27 – 2.13, *p*=0.810 | Contrast=-3.46,  95% CI: -7.39 – 0.46, *p*=0.249 |
| **SCAT Symptom Severity** | χ^2^=1.98, *p*=0.160 | **χ^2^=66.7, *p*<0.001** | **χ^2^=10.6, *p*=0.014** | Males | **Contrast=13.4,**  **95% CI: 4.87 – 22.0, *p*=0.006** | Contrast=6.45,  95% CI: -1.42 – 14.3, *p*=0.216 | Contrast=4.47,  95% CI: -5.59 – 14.5, *p*=0.384 |
|  |  |  |  | Females | Contrast=-0.59,  95% CI: -9.07 – 7.90, *p*=0.892 | Contrast=-4.99,  95% CI: -12.2 – 2.25, *p*=0.354 | Contrast=-7.85,  95% CI: -17.0 – 1.28, *p*=0.276 |
| **SAC Score** | χ^2^=1.62, *p*=0.203 | **χ^2^=43.9, *p*<0.001** | χ^2^=2.89, *p*=0.409 | Males | Contrast=-1.03,  95% CI: -2.19 – 0.13, *p*=0.243 | Contrast=0.48,  95% CI: -12.7 – 13.6, *p*=0.943 | Contrast=0.67,  95% CI: -12.6 – 14.0, *p*=0.999 |
|  |  |  |  | Females | **Contrast=-2.20,**  **95% CI: -3.25 – -1.15, *p*<0.001** | Contrast=-0.20,  95% CI: -12.9 – 12.5, *p*=0.975 | Contrast=0.76,  95% CI: -12.4 – 13.9, *p*=0.999 |
| **Serum NfL** | **χ^2^=20.5, *p*<0.001** | χ^2^=4.49, *p*=0.21 | **χ^2^=17.9, *p*<0.001** | Males | Contrast=1.52,  95% CI: -0.17 – 3.20, *p*=0.078 | **Contrast=4.30,**  **95% CI: 1.21 – 7.38, p=0.012** | **Contrast=8.64,**  **95% CI: 3.42 – 13.9, *p*=0.003** |
|  |  |  |  | Females | Contrast=-1.70,  95% CI: -3.50 – 0.10, *p*=0.128 | Contrast=-1.04,  95% CI: -2.07 – -0.01, *p*=0.141 | Contrast=-0.93,  95% CI: -2.91 – 1.05, *p*=0.357 |
| **Serum Tau** | χ^2^=0.10, *p*=0.75 | χ^2^=1.77, *p*=0.62 | χ^2^=6.98, *p*=0.07 | Males | Contrast=-0.03,  95% CI: -0.15 – 0.09, *p*=0.999 | Contrast=0.03,  95% CI: -0.11 – 0.15, *p*=0.778 | **Contrast=0.23,**  **95% CI: 0.05 – 0.42, *p*=0.039** |
|  |  |  |  | Females | Contrast=-0.05,  95% CI: -0.21 – 0.11, *p*=0.999 | Contrast=0.03,  95% CI: -0.35 – 0.42, *p*=0.862 | Contrast=-0.03,  95% CI: -0.20– 0.13, *p*=0.999 |
| **Serum UCHL1** | **χ^2^=4.23, *p*=0.040** | χ^2^=3.03, *p*=0.39 | χ^2^=1.89, *p*=0.60 | Males | Contrast=-0.39,  95% CI: -2.59 – 1.81, *p*=0.728 | Contrast=2.55,  95% CI: -4.17 – 9.27, *p*=0.914 | Contrast=10.0,  95% CI: -9.87 – 30.0, *p*=0.969 |
|  |  |  |  | Females | Contrast=-1.14,  95% CI: -4.91 – 2.64, *p*=0.999 | Contrast=-0.81,  95% CI: -4.50 – 2.89, *p*=0.669 | Contrast=2.21,  95% CI: -3.34 – 7.76, *p*=0.999 |
| **Serum GFAP** | χ^2^=0.430, *p*=0.51 | **χ^2^=11.2, *p*=0.011** | **χ^2^=8.13, *p*=0.043** | Males | **Contrast=20.3,**  **95% CI: 9.66 –30.9, *p*<0.001** | Contrast=26.4,  95% CI: 1.10 –51.7, *p*=0.082 | Contrast=6.62,  95% CI: -3.26 – 14.5, *p*=0.215 |
|  |  |  |  | Females | Contrast=0.73,  95% CI: -27.9 – 29.4, *p*=0.96 | Contrast=-10.6,  95% CI: -30.6 – 9.48, *p*=0.903 | Contrast=-10.3,  95% CI: -32.9 – 12.4, *p*=0.748 |

**Supplementary Table 2.** Baseline comparisons between non-SRC control footballers and SRC footballers

|  |  | **Non-SRC baseline (Mean)** | **SRC baseline (Mean)** | ***p*-value** |
| --- | --- | --- | --- | --- |
| **Serum NfL** | Males | 4.97 pg/mL | 5.75 pg/mL | *p*=0.158 |
|  | Females | 5.16 pg/mL | 5.94 pg/mL | *p*=0.298 |
| **Serum Tau** | Males | 0.34 pg/mL | 0.39 pg/mL | *p*=0.573 |
|  | Females | 0.61 pg/mL | 0.42 pg/mL | *p*=0.108 |
| **Serum UCHL1** | Males | 11.67 pg/mL | 7.04 pg/mL | *p*=0.432 |
|  | Females | 11.86 pg/mL | 4.47 pg/mL | *p*=0.120 |
| **Serum GFAP** | Males | 57.36 pg/mL | 47.52 pg/mL | *p*=0.258 |
|  | Females | 63.41 pg/mL | 70.68 pg/mL | *p*=0.852 |

**Supplementary Table 3.** Serum biomarker comparisons of non-concussed and concussed Australian footballers.

|  | **Area under the receiver operating characteristics (AUROCs), *p*-value** | | | |
| --- | --- | --- | --- | --- |
|  |  | **Control vs 2-days** | **Control vs 6-days** | **Control vs 13-days** |
| **Serum NfL** | Males | **AUROC=0.73, *p*=0.018** | **AUROC=0.85**, ***p*<0.001** | **AUROC=0.79**, ***p*=0.002** |
|  | Females | AUROC=0.63, *p*=0.418 | AUROC=0.55, *p*=0.753 | AUROC=0.52, *p*=0.908 |
| **Serum Tau** | Males | AUROC=0.51, *p*=0.937 | AUROC=0.55, *p*=0.584 | **AUROC=0.72**, ***p*=0.022** |
|  | Females | AUROC=0.59, *p*=0.563 | AUROC=0.64, *p*=0.345 | AUROC=0.66, *p*=0.298 |
| **Serum UCHL1** | Males | AUROC=0.61, *p*=0.254 | AUROC=0.56, *p*=0.564 | AUROC=0.59, *p*=0.354 |
|  | Females | AUROC=0.66, *p*=0.298 | AUROC=0.73, *p*=0.115 | AUROC=0.75, *p*=0.105 |
| **Serum GFAP** | Males | AUROC=0.60, *p*=0.303 | AUROC=0.52, *p*=0.796 | AUROC=0.54, *p*=0.682 |
|  | Females | AUROC=0.54, *p*=0.817 | AUROC=0.53, *p*=0.834 | AUROC=0.52, *p*=0.908 |


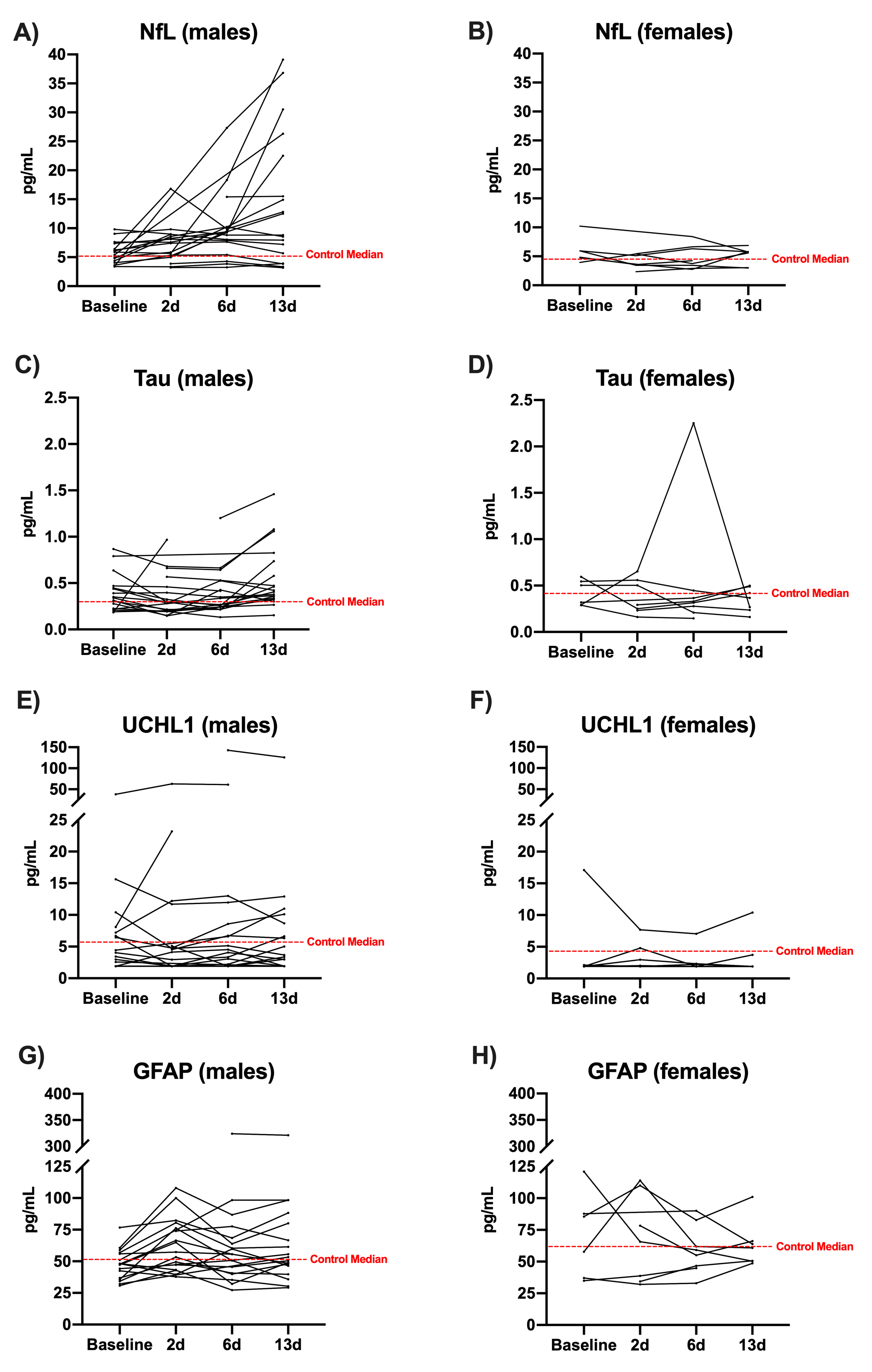


**Supplementary Figure 1.** Temporal profile of serum biomarkers pre- and post-concussion.
